# Spatial distribution and incidence of HIV/AIDS cases in Santa Cruz do Sul, state of Rio Grande do Sul, 2001 to 2020

**DOI:** 10.1590/S2237-96222022000300020

**Published:** 2022-12-19

**Authors:** Priscila Braga Rosa, Daniel Felipe Schroeder, Camilo Darsie, Micila Chielle, Marlua Luiza Kuhl Pontel, Gabriela de Borba Correa, Ivinildo José Vilichane, Tiago Antônio Heringer, Jane Dagmar Pollo Renner, Lia Gonçalves Possuelo

**Affiliations:** 1 Universidade de Santa Cruz do Sul, Departamento de Ciências da Vida, Santa Cruz do Sul, RS, Brazil; 2 Universidade de Santa Cruz do Sul, Programa de Pós-graduação em Educação, Santa Cruz do Sul, RS, Brazil; 3 Universidade de Santa Cruz do Sul, Programa de Pós-Graduação em Promoção da Saúde, Santa Cruz do Sul, RS, Brazil; 4 Centro Municipal de Atendimento à Sorologia, Santa Cruz do Sul, RS, Brazil; 5 Hospital Provincial de Xai-xai, Gaza, Moçambique

**Keywords:** Population Spatial Distribution, Access to Treatment, HIV, Incidence, Epidemiology, Descriptive

## Abstract

**Objective:**

to describe the spatial distribution, treatment status and characteristics of cases of people infected with the human immunodeficiency virus HIV in Santa Cruz do Sul, RS, 2001 to 2020.

**Methods:**

descriptive study with data from individuals undergoing treatment for HIV/AIDS, in Santa Cruz do Sul, diagnosed from January 2001 to October 2020.

**Results:**

708 (94.4%) cases were analyzed, of these (58.2%) were male, with a mean age of 39 years, the maximum incidence rate was in 2019 (59.4/100,000 inhabitants), there was a high frequency of cases in the south and central region of the city, 92.9% of these individuals were still in active treatment and the dropout rate was 7.1% in the period.

**Conclusion:**

a higher incidence of HIV was observed in adult male, from the central and southern regions of the city, with a treatment rate close to the goals of the World Health Organization and a low dropout rate.

**Study contributions**

**Main results**

The average incidence of HIV cases was 27.6/100,000 inhabitants, with the minimum rate in 2004 (3.4/100,000 inhabitants) and the maximum rate in 2019 (59.4/100,000 inhabitants).The cases were more frequent among males and in the south and central regions of the municipality.

**Implications for services**

The upward trend in the data points to the need to optimize public health actions, through the Department of Health, aimed at the most affected population, seeking to improve these indicators.

**Perspectives**

The development of a permanent health education program for workers and the intensification of health education strategies for the population may contribute to the qualification of HIV indicators in the municipality.

## INTRODUCTION

Human immunodeficiency virus (HIV), the causative agent of acquired immunodeficiency syndrome (AIDS), remains as a global public health problem, having accounted for more than 36 million deaths worldwide.^1^ ^,^ ^2^ At the end of 2020, about 38 million people were living with HIV, of whom more than two-thirds were individuals living in Africa. In that same year, 680,000 people died of HIV-related causes, and 1.5 million people contracted the infection.^3^ ^,^ ^4^

In Brazil, in 2019, 41,909 new cases of HIV and 37,308 cases of AIDS were diagnosed, most of them in the Southeast region (35.3%), followed by the Northeast (25.6%), South (18.2%), North (11.8%) and Midwest (9.1%) regions. Between 1980 and 2020, more than 1 million cases of AIDS were diagnosed in the country, and the AIDS detection rate, which was 21.9/100,000 inhabitants in 2012 reduced to 17.8/100,000 inhabitants in 2019, corresponding to an 18.7% decrease.^1^

From the beginning of the AIDS epidemic in 1980 to December 31, 2019, 349,784 HIV/AIDS- related deaths were reported in Brazil. In the municipality of Santa Cruz do Sul, state of Rio Grande do Sul, in the same period, a total of 939 cases of HIV/AIDS were reported on the Notifiable Health Condition Information System (*Sistema de Informação de Agravos de Notificação* - SINAN), with 248 deaths registered from 1996 to 2019.^1^

The Joint United Nations Program on HIV/AIDS (UNAIDS) launched, in 2014, the ambitious and challenging strategy to end the AIDS epidemic by 2030. Among the actions discussed, it was defined that AIDS will no longer be a public health threat by 2030. In order to achieve the new global targets proposed by the program (95-95-95 targets), it is necessary to redouble efforts to reduce the number of cases and annual HIV-related deaths.^2^ ^-^ ^4^

In 2014, the Ministry of Health published the update of the Clinical Protocol and Therapeutic Guidelines (*Protocolo Clínico e Diretrizes Terapêuticas* - PCDT), which introduced antiretroviral therapy (ART), a treatment as prevention (TasP) and expanded treatment for all people living with HIV/AIDS, regardless of the stage of infection, via the public health systems network, in addition to the introduction of post-exposure prophylaxis (PEP).^5^ All these measures corroborate the targets proposed by UNAIDS aimed at ending AIDS as a public health threat.^3^

Social vulnerability is among the factors related to the emergence of new HIV cases, an increasingly comprehensive concept that interconnects individual and collective aspects. It is a determining factor in the form of care during the health-disease process, in which a poorer and less knowledgeable population ends up being more affected by several problems, such as HIV.^6^ ^,^ ^7^

Surveillance strategies used to control HIV should include reliable information systems and efficient methods for locating cases.^7^ In this context, the use of geoprocessing techniques assists in understanding the geographical distribution of diseases, such as HIV, and can help in the identification of associated risk factors and in the analysis of critical points of dissemination, which contributes to the conception, planning and allocation of health resources for prevention and treatment.^8^

Thus, this study aimed to describe the spatial distribution, treatment status and epidemiological characteristics of cases of individuals infected with HIV/AIDS in the municipality of Santa Cruz do Sul, state of Rio Grande do Sul, between 2001 and 2020.

## METHODS

This was a descriptive study, in which data on people undergoing treatment for HIV/AIDS at the Centro Municipal de Atendimento a Sorologia (CEMAS), living in the municipality of Santa Cruz do Sul, state of Rio Grande do Sul, diagnosed in the period from January 2001 to October 2020, were included.

The municipality of Santa Cruz do Sul, with an estimated population of 131.365 inhabitants, in 2020, is one of the main centers of German colonization in the state of Rio Grande do Sul. It is located in Vale do Rio Pardo region, on the lower Northeast slope of the state, 155 km from Porto Alegre, the capital city. It has a territorial unit area of 733.898 km². The Municipal Human Development Index (MHDI) was 0.773, in 2010, ranking in 26^th^ in the state. The gross domestic product (GDP) *per capita*, for the year 2017, was BRL 64,653.78.^9^

CEMAS is a specialized service that provides care for cases of HIV/AIDS and other sexually transmitted infections (STIs). It has two services: the Counseling and Testing Center (CTC), which performs STI testing and develops self-care prevention programs; and the Specialized Assistance Service (SAS), which provides specialized care and follow-up to people already diagnosed. CEMAS provides care for all residents in the municipality of Santa Cruz do Sul, both individuals who use the public health service and those who use the private healthcare. People who use the private health service have medical visits at CEMAS aiming at obtaining medication, and all of them have medical records for follow-up care.

The physical medical records obtained from CEMAS were used as data source. We included confirmed cases of HIV in individuals aged 18 years and older, followed at CEMAS and living in the municipality of Santa Cruz do Sul at the time of diagnosis, between January 2001 and October 2020. HIV cases among homeless people and those with incomplete data in their medical records were excluded. Cases were selected according to the notification period, taking into account the PCDT update in 2014: from 2001 to 2013 (pre-PCDT period); and from 2014 to 2020 (post-PCDT period).

We considered as treatment dropout, non-attendance among users at the health service for three months after obtaining their medications or non-attendance to medical consultations in an interval greater than six months.^10^ ^,^ ^11^

The data, collected from medical records by the CEMAS team - duly trained by the researchers for this collection - were passed on to the researchers anonymously, with no possibility of identifying the subjects included in the study. Data were accessed by the health unit team between May and June, 2021.

The variables studied were: sex (male and female), age group (16-35, 36-59, 60 years and older), neighborhood of residence (30 neighborhoods), year of diagnosis (2001 to 2013, pre-PCDT period; and from 2014 to 2020, post-PCDT period), and treatment follow-up (dropout or active case).

Data were tabulated in a spreadsheet, using the Excel program, with calculation of mean and standard deviation of the variables. Descriptive analyses of absolute and relative frequencies were performed, regarding the characteristics of the cases. The calculation of the average incidence rate in the period studied was done by dividing the total number of new HIV cases per year, by the total resident population estimated for the year 2010, multiplied by 100,000. The calculation of HIV incidence per neighborhood was done by dividing the total number of new cases for each period of the study (2001 to 2013, and 2014 to 2020), by the estimated population in 2010 and 2019,^9^ respectively, multiplied by 100,000.

The spatial distribution of HIV cases was described based on the information of the neighborhood of residence. Initially, in order to verify the distribution of HIV cases, they were grouped into two periods, according to the year of notification: from 2001 to 2013 (pre-PCDT period) and from 2014 to 2020 (post-PCDT period). The thematic maps were created using the QGIS 3.14.15 software, version “Pi”, available free of charge. Vector bases of the urban area of the municipality and neighborhoods were obtained from the geoprocessing sector of the municipality of Santa Cruz do Sul. For organization and division of cutoff points in the incidence scale by neighborhoods, five levels were created, namely: ZERO; 1 to 150; 151 to 300; 301 to 500; over 500. Warmer color tones represented the highest incidence rates of cases. The maps were created on the SIRGAS 2000 Coordinate Reference System (CRS), Brazil’s official standard. In the spatial distribution analysis, only cases of people living in urban areas of the municipality were included. Trend and correlation analyses were performed by means of Pearson correlation, using the GraphPad Prism software, version 6.0.

Taking into consideration Resolution No. 499/2012 of the National Health Council (*Conselho Nacional de Saúde* - CNS), this study was approved by the Research Ethics Committee of the Universidade de Santa Cruz do Sul, opinion No. 4,662,011 and the Certificate of Submission for Ethical Appraisal No. 41768721.5.0000.5343, on April 21, 2021.

## RESULTS

A total of 750 new cases of HIV were registered during the study period, of which 42 (5.6%) were excluded, because they presented inconsistencies in their registration or had no fixed residence, totaling 708 (94.4%) cases. A total of 21 cases (2.8%) lived in rural areas of the municipality and were not included in the spatial distribution analysis. Among the 708 cases, 58.2% were male, and the average age was 39 years old (standard deviation ± 12.5), ranging from 18 to 84 years old. The most frequent age group was 36 to 59 years old (47.7%). It could be seen that 92.9% of people were undergoing active treatment and 7.1% (n = 50) had dropped outr treatment. When comparing the analyzed periods, a higher proportion of males diagnosed in the post-PDCT period (p-value < 0.001) was found, (Table 1).

**Table 1:** - Characteristics of people living with HIV/AIDS in Santa Cruz do Sul, state of Rio Grande do Sul, Brazil, before and after the PCDT update, 2001 to 2020

Characteristics	Pre-PCDT^a^ n (%)	Pos-PCDT^b^ n (%)	Total (%)	p-value^c^
Sex				0.002
Female	126 (49.2)	170 (37.6)	296 (41.8)	
Male	130 (50.8)	282 (62.4)	412 (58.2)	
Age group (in years)				0.310
16-35	125 (48.8)	194 (42.9)	319 (45.1)	
36-59	114 (44.5)	224 (49.6)	338 (47.7)	
60 and older	17 (6.7)	34 (7.5)	51 (7.2)	
Treatment				0.380
Active	235 (95.2)	422 (93.4)	657 (92.9)	
Abandonment	21 (0.8)	29 (6.6)	50 (7.1)	

a) Pre-PCDT (2001-2013); b) Post-PCDT (2014-2020); c) Pearson’s chi-squared test.

Legend: HIV/AIDS = Human immunodeficiency virus/acquired immunodeficiency syndrome; PCDT = *Protocolo Clínico e Diretrizes Terapêuticas* - Clinical Protocol and Therapeutic Guidelines.

**Figure 1:**
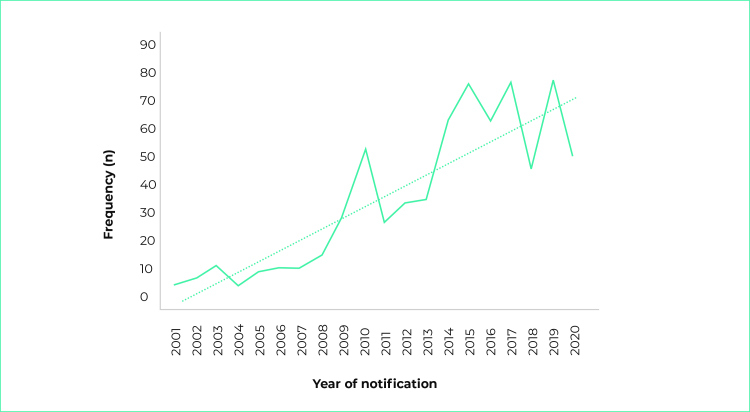
- Frequency of HIV/AIDS cases, by year of notification, Santa Cruz do Sul, state of Rio Grande do Sul, Brazil, 2001 to 2020

Note: The dotted little line indicates trend curve; p-value < 0.001 (Pearson correlation).

The average HIV incidence rate during the study period was 27.6/100,000 inhabitants, with the lowest rate in 2004 (3.4/100,000 inhabitants), and the highest rate in 2019 (59.4/100,000 inhabitants), with an upward trend line (Figure 1).

**Figure 2:**
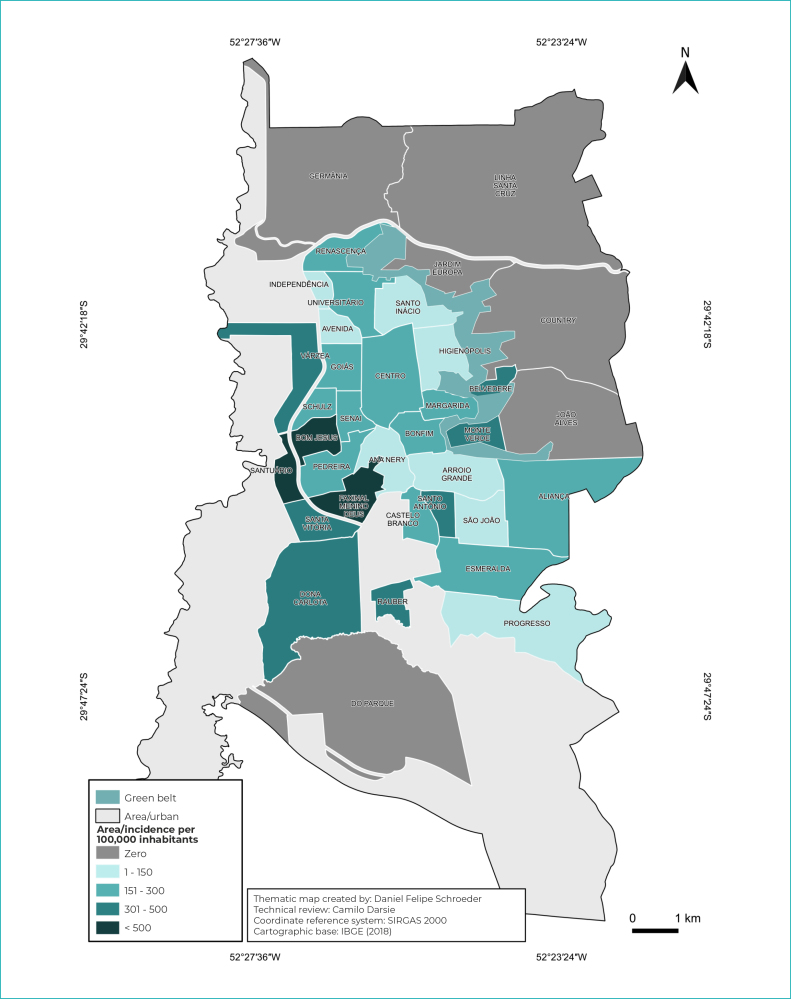
- Incidence of HIV/AIDS cases in Santa Cruz do Sul, state of Rio Grande do Sul, Brazil, 2001 to 2013

**Figure 3:**
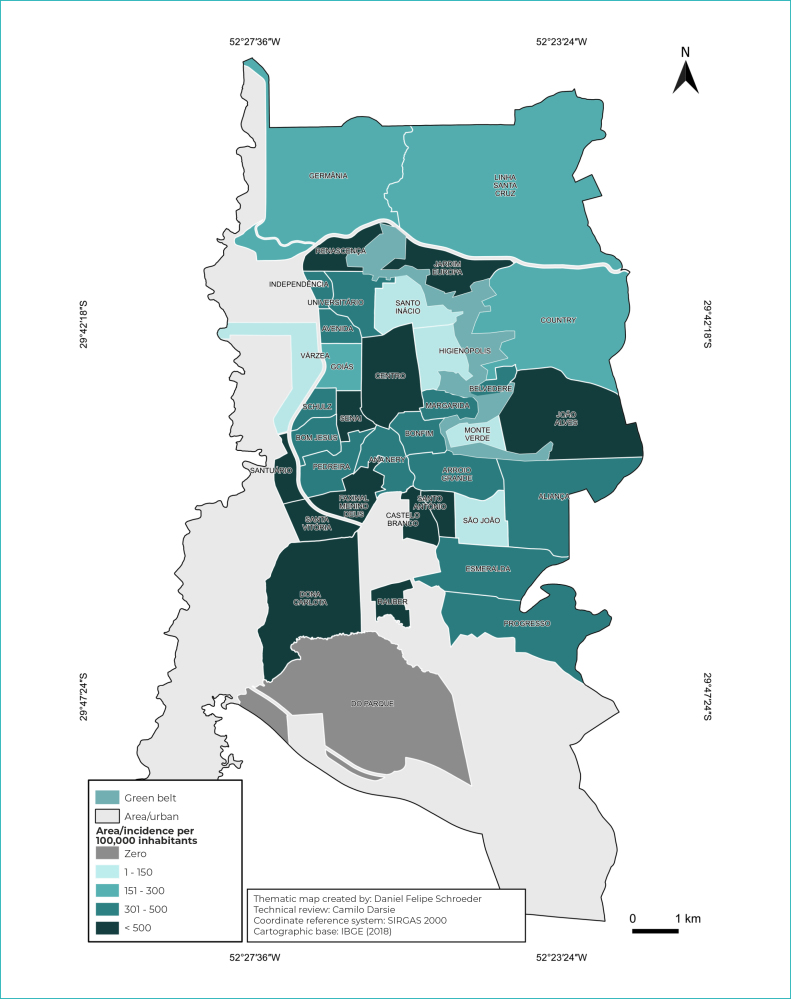
- Incidence of HIV /AIDS cases between 2014 and 2020, in the municipality of Santa Cruz do Sul, state of Rio Grande do Sul, Brazil

Spatial analysis showed that, until 2013, the highest incidences of HIV/AIDS were concentrated in the south region of the municipality, which has low socioeconomic levels. As of 2014, a higher incidence of HIV/AIDS cases was observed in the southern and central regions of the municipality (Figures 2 and 3).

## DISCUSSION

Data show a higher incidence of HIV in adult male living in areas of low socioeconomic development, with a treatment rate close to the WHO goals, low abandonment rate and increasing incidence trend. A higher proportion of cases diagnosed among male was observed in the post-PCDT period, with greater dispersion in the central and southern areas of the municipality, also in the post-PCDT period.

The data also showed disparity in the distribution of cases, with cases being predominant in male, as described in other national studies.^12^ ^,^ ^13^ A study conducted in the state of Rio Grande do Norte concluded that most HIV cases were reported among males, corroborating studies conducted in the state of Mato Grosso do Sul.^14^ This characteristic is a possible reflection of cultural, economic and social factors related to exposure. Gender issues impose themselves especially on relationships between men and women and on relationships between men and other men, due to labels they have been given based on "hegemonic masculinity", and practices such as multiple sexual partnership, illicit drug use and alcohol consumption may contribute to vulnerability to HIV.^15^

The predominant age group was 36 to 59 years old, and the average age was 39 years old. According to data from the Ministry of Health, this age range is just above that described for Brazil (25 to 39 years old). From 2001 to 2015, in the state of Rio Grande do Sul, the highest detection rates were identified in the 30 to 39 age group.^16^ The increase in the number of individuals aged 50 years and older with AIDS, in Brazil and worldwide, is a fact that can be evaluated based on demographic data, analyzing the increase in the number of notifications and the aging population with the disease.^17^ In a study conducted in the state of Piauí, regarding the age group of those infected with HIV/AIDS, the 31 and 50 age group (58.2%) was the most reported, fitting into this category the individuals with chronic HIV infection and an active sex life.^18^

A total of 7.1% of the people evaluated in this study dropped out treatment. This rate is similar to that found in another study conducted between 2007 and 2016, in the state of Rondônia (10.5%).^18^ Another study, conducted in the state of Amapá, identified 21.5% of ART dropped out among non-pregnant women, and the following factors that corroborate dropped out were highlighted: maintenance of the initial regimen (73.7%), registration of individuals with outdated viral load (74.5%), presence of psychiatric disorders (18%), lack of affective social support (12.3%) and difficulties in attending the Specialized Assistance Service (SAS) to obtain ART (26%).^19^

In this context, in 2009, the Ministry of Health established norms and criteria that provide guidance for health professionals related to individuals who dropped out treatment. The programmatic manuals emphasize that, for a better prognosis and treatment, strict adherence to ART is necessary, given that irregularities when taking the medication or its abandonment increase the probability of HIV replication, as well as the spread of multidrug-resistant virus.^20^

In this study, the frequency of people undergoing antiretroviral therapy reached 92.9% and according to UNAIDS 95-95-95 target, by 2030, 95% of all people living with HIV will know they have the virus, 95% of all people with diagnosed HIV infection will receive antiretroviral therapy uninterruptedly, and 95% of all people receiving antiretroviral therapy will have viral suppression. Mathematical models suggest that achieving these goals will enable the world to end the AIDS epidemic by 2030, generating substantial health and economic benefits.

In the period from 2001 to 2020, it could be seen a higher incidence of HIV/AIDS cases in the south zone of the urban area of the municipality, where families with low purchasing power, income below 5 minimum wages (MW) and high social vulnerability, live. Studies conducted in the municipality, between 2012 and 2018, showed a high incidence of tuberculosis and hepatitis C in the same region, demonstrating that the socioeconomic factor is an aggravating factor for the transmission of infectious and contagious diseases in the region.^22^ ^,^ ^23^ A study conducted in Belém, state capital of Pará, corroborates the data of the present study, which showed that individuals with monthly income less than or equal to 2 MWs, homeless people with a chemical dependency, precarious housing conditions, poor diet, low income, lack of basic sanitation and low level of education constitute determinant elements related to the increase in the incidence of HIV/AIDS in communities.^24^

The variation in the incidence between the two periods analyzed (3.4/100,000 *vs*. 59.4/100,000), may be related to the PCDT update, in addition to the inclusion of HIV in the list of compulsorily notifiable health conditions, on July 6, 2014 (Ordinance MS No. 1,271).^25^

In the period from 2014 to 2020, there was an increase in the dispersion of the number of cases, which were mainly concentrated in the central and southern areas of the municipality. According to a study conducted in the municipalities of the state of Rondônia, the central region of the state also showed a greater dispersion of the number of cases.^24^ Among the spatial determinants are: high population density, regions with the highest poverty levels, and border areas with a higher proportion of migrants, which function as large corridors of movement of people, including truck drivers and sex workers.^25^

The 2030 Agenda for Sustainable Development reflects the interdependence and complexity of a changing world that requires global collective action. The response to AIDS is no exception: the epidemic will not end without addressing the determinants of health and vulnerability, as well as the holistic needs of people at risk of HIV infection and living with the virus. People living with HIV are often in fragile communities and are most affected by discrimination, inequality and instability. Their concerns must be at the center of sustainable development efforts.^2^

The limitation of this study is inherent to research with secondary data, which refers to the quality and coverage of medical records with possible occurrence of incompleteness of these records, which may eventually cause some distortion in the analysis of the spatial distribution of cases. However, its comprehensive character and the possibility of replicating the methodology in other geographical areas and periods stand out, in addition to the fact that the results obtained are adequate for the orientation of actions aimed at disease prevention and control.

Given this scenario, the mapping of cases and epidemiological analysis provide significant support for the characterization of the spatial distribution of HIV, aiming to improve the action of public policies regarding prevention and health education. Taking into account the increasing incidence of cases in the municipality of Santa Cruz do Sul in the last two decades, further studies are needed to monitor the situation of affected individuals, in order to investigate the reasons that lead to treatment dropout and promote health education and follow-up programs for the population living in the most affected regions, in order to raise awareness about the severity of this disease and its impacts.
